# Increasing polypharmacy - an individual-based study of the Swedish population 2005-2008

**DOI:** 10.1186/1472-6904-10-16

**Published:** 2010-12-02

**Authors:** Bo Hovstadius, Karl Hovstadius, Bengt Åstrand, Göran Petersson

**Affiliations:** 1 eHealth Institute and School of Natural Sciences, Linnaeus University, SE-391 82 Kalmar, Sweden; 2 Department of Statistics, Uppsala University, SE-751 01 Uppsala, Sweden; 3 eHealth Institute and School of Health and Caring Sciences, Linnaeus University, SE-391 82 Kalmar, Sweden

## Abstract

**Background:**

An increase in the use of drugs and polypharmacy have been displayed over time in spite of the fact that polypharmacy represents a well known risk factor as regards patients' health due to the adverse drug reactions, drug-drug interactions, and low adherence to drug therapy arising from polypharmacy. For policymakers, as well as for clinicians, it is important to follow the developing trends in drug use and polypharmacy over time. We wanted to study if the prevalence of polypharmacy in an entire national population has changed during a 4-year period.

**Methods:**

By applying individual-based data on dispensed drugs, we have studied all dispensed prescribed drugs for the entire Swedish population during four 3-month periods 2005-2008. Five or more (DP ≥5) and ten or more (DP ≥10) dispensed drugs during the 3-month period was applied as the cut-offs indicating the existence of polypharmacy and excessive polypharmacy respectively.

**Results:**

During the period 2005-2008, the prevalence of polypharmacy (DP≥5) increased by 8.2% (from 0.102 to 0.111), and the prevalence of excessive polypharmacy (DP≥10) increased by 15.7% (from 0.021 to 0.024).

In terms of age groups, the prevalence of polypharmacy and excessive polypharmacy increased as regards all ages with the exception of the age group 0-9 years. However, the prevalence of excessive polypharmacy displayed a clear age trend, with the largest increase for the groups 70 years and above. Furthermore, the increase in the prevalence of polypharmacy was, generally, approximately twice as high for men as for women. Finally, the mean number of dispensed drugs per individual increased by 3.6% (from 3.3 to 3.4) during the study period.

**Conclusions:**

The prevalence of polypharmacy and excessive polypharmacy, as well as the mean number of dispensed drugs per individual, increased year-by-year in Sweden 2005-2008.

## Background

It is clear that a continuous increase in the overall level of drug use, especially among elderly, has been noted in several countries. Moreover, an increase in the number of individuals experiencing polypharmacy, ie. the concurrent use of several different drugs, has also been reported [1-3].

Whilst the use of a number of different drugs for many individuals appears to be a rational drug therapy, and polypharmacy is assumed to provide major health benefits for the well being of large groups of individuals suffering from different diseases, polypharmacy is also a well known risk factor due to adverse drug reactions, drug-drug interactions, and low adherence to drug therapy [3-5].

In addition, it is also assumed that polypharmacy causes unnecessary health expenditure [5], directly due to redundant drug sales and indirectly due to the increased level of hospitalization caused by drug-related problems [6]. Drug-related problems are reported to cause a substantial proportion of all emergency treatment and admissions to hospitals as regards elderly patients [4,7]. Consequently, there have been many attempts to reduce the number of prescribed drugs to individuals experiencing polypharmacy, especially as regards the elderly [1].

Furthermore, it should also be noted that previous studies of polypharmacy have primarily been conducted on samples of elderly individuals admitted to hospitals or nursing homes [5,8]. Only a few studies have been based on population-based information [5,9,10], and some of these studies have also been limited to elderly individuals [11-14]. A recent register study showed that 2/3 of all individuals in a national population who were being prescribed with 5 or more drugs were < 70 years of age [15], indicating that multiple medication use is not only relevant as regards elderly individuals.

Clearly, for policymakers, as well as for clinicians, it is important to follow the developing trends in drug use and polypharmacy over time, and not only in the elderly age groups but also for the large number of middle-aged individuals subject to polypharmacy. In this context, the establishment of the Swedish Prescribed Drug Register in 2005 made it possible to apply individual data in exploring and analyzing the utilization of polypharmacy in an entire national population. Such individual-based data may also be applied in longitudinal studies of the development of drug use.

### Aim of the study

We wanted to study if the prevalence of polypharmacy in an entire national population has changed during a 4-year period.

## Methods

By using individual based data on dispensed drugs, we studied all dispensed prescribed drugs for the entire Swedish population during four 3-month periods (July, August and September) 2005-2008. These data were extracted from the Swedish Prescribed Drug Register [10].

In this study, the prevalence of polypharmacy was defined as the proportion of individuals receiving five or more dispensed prescription drugs (DP≥5) during a 3-month period.

As a definition of excessive polypharmacy, we applied ten or more dispensed drugs (DP≥10) for an individual during the study period [12]. Consequently, the prevalence of excessive polypharmacy was defined as the proportion of individuals receiving ten or more dispensed drugs during a 3-month period. As five or more dispensed drugs comprises the most commonly applied definition of polypharmacy [8,12,16] and 10 or more dispensed drugs is the most widely used definition of "excessive" polypharmacy [9,12,16,17] our definitions are intended to enable comparisons with other studies.

The development of the prevalence of drug use defined as the proportion of individuals with one or more dispensed drugs (DP≥1) during a 3-month period is illustrated for purpose of comparison.

### The Swedish Prescribed Drug Register

The Swedish Prescribed Drug Register covers the entire Swedish population and includes approximately 82% of all Defined Daily Doses (DDD) dispensed in Sweden. The register does not include data on OTC medications (13%), in-hospital medications (4%), and non-institutional care medications (1% of all DDD distributed in Sweden). This register is not complete as regards vaccines or for non-dose-dispensed drugs in nursing homes.

The Swedish Prescribed Drug Register is individual-based and contains data from dispensed out-patient prescriptions at all Swedish pharmacies from July 1, 2005. The registration of dispensed drugs is mandatory and the following data from the register was used in our study: dispensed drug (substance), date of dispensing, age, gender, and a unique identifier (personal identification number) of the patient.

All processing of the individual data of dispensed drugs in our study was undertaken anonymously, without the original personal identification number. Instead, a unique temporary individual identifier, specifying gender and year of birth, was applied and the study population was stratified by gender and age (10-year classes). The results of our study were presented with respect to the number of individuals per gender and age group in the Swedish population during the corresponding periods.

Also, the values applied were the number of individuals and the number of dispensed prescription drugs per individual, and the definition of drug was the chemical entity or substance comprising the fifth level in the Anatomical Therapeutic Chemical (ATC) classification system.

Calculation of sums and frequencies were aggregated using Microsoft Excel (version 5.1.26).

The study was approved by the Regional Ethical Review Board in Linköping, Sweden.

## Results

### The development of polypharmacy

The prevalence of polypharmacy (DP≥5) in the entire population increased by 8.2% (from 0.102 to 0.111) 2005-2008 (Table 1). The number of individuals with DP≥5 increased by 10.4% (from 922,949 to 1,019,324) (Table 2).

**Table 1:** The prevalence of dispensed drugs, polypharmacy, and excessive polypharmacy

Age group	DP≥1				DP≥5				DP≥10			
			
	2005	2006	2007	2008	2005	2006	2007	2008	2005	2006	2007	2008
0-9	0.183	0.194	0.184	0.188	0.005	0.005	0.005	0.005	0.000	0.000	0.000	0.000
10-19	0.214	0.223	0.228	0.238	0.008	0.008	0.008	0.008	0.000	0.000	0.000	0.001
20-29	0.314	0.313	0.308	0.304	0.016	0.017	0.017	0.017	0.001	0.001	0.001	0.001
30-39	0.332	0.337	0.335	0.333	0.029	0.029	0.029	0.030	0.003	0.003	0.003	0.004
40-49	0.390	0.396	0.395	0.394	0.053	0.054	0.055	0.056	0.008	0.008	0.009	0.009
50-59	0.533	0.536	0.537	0.536	0.113	0.115	0.117	0.119	0.019	0.020	0.020	0.021
60-69	0.640	0.649	0.657	0.662	0.197	0.202	0.209	0.214	0.037	0.039	0.040	0.041
70-79	0.784	0.789	0.794	0.797	0.350	0.359	0.367	0.376	0.078	0.083	0.085	0.088
80-89	0.833	0.843	0.849	0.853	0.475	0.492	0.504	0.514	0.121	0.139	0.143	0.147
90-	0.780	0.790	0.790	0.795	0.494	0.524	0.524	0.532	0.126	0.159	0.160	0.162
Total	0.426	0.433	0.433	0.436	0.102	0.105	0.108	0.111	0.021	0.022	0.023	0.024

The prevalence of dispensed drugs (DP≥1), polypharmacy (DP≥5), and excessive polypharmacy (DP≥10) in different age groups in Sweden 2005-2008. n = 9,029,750 (2005), 9,080,505 (2006) 9,148,092 (2007) and 9,219,637 (2008).

**Table 2:** Number of individuals with polypharmacy, and excessive polypharmacy

Age group	DP≥5				DP≥10			
		
	2005	2006	2007	2008	2005	2006	2007	2008
0-9	4,698	4,829	4,529	4,653	243	235	246	230
10-19	9,082	8,974	9,300	9,693	579	578	574	620
20-29	17,598	17,993	18,481	18,930	1,486	1,598	1,601	1,646
30-39	35,926	36,118	35,857	36,297	4,129	4,312	4,230	4,345
40-49	64,044	66,952	69,170	70,883	9,666	10,058	10,730	10,919
50-59	137,191	137,651	138,873	139,519	22,785	23,587	23,763	24,142
60-69	191,510	205,066	220,723	234,603	36,053	39,159	42,206	44,942
70-79	231,413	236,654	242,252	250,348	51,575	54,790	56,488	58,788
80-89	195,172	203,814	208,939	213,207	49,833	57,490	59,282	61,060
90-	36,315	39,228	39,985	41,191	9,269	11,884	12,197	12,552
Total	922,949	957,279	988,109	1,019,324	185,618	203,691	211,317	219,244

Number of individuals with polypharmacy (DP≥5), and excessive polypharmacy (DP≥10) in different age groups in Sweden 2005-2008.

**Figure 1:**
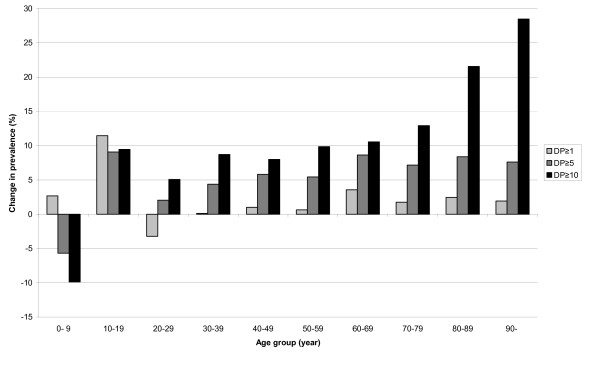
**The change in prevalence of dispensed drugs, polypharmacy, and excessive polypharmacy**. The change (%) in prevalence of dispensed drugs (DP≥1), polypharmacy (DP≥5), and excessive polypharmacy (DP≥10) in different age groups in Sweden during 2005-2008.

The prevalence increased in all age groups, except for the age group 0-9 years and the largest increase in the prevalence was in the age group 10-19 with an increase of 9.1%. In the age groups 60-69 to 90-years, the increase was between 7.2% and 8.6% (Figure 1).

**Figure 2:**
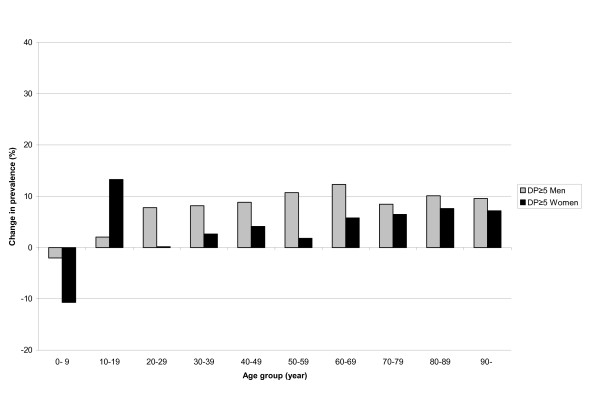
**The change in prevalence of polypharmacy for men and women**. The change (%) in prevalence of polypharmacy (DP≥5) for men and women in different age groups in Sweden 2005-2008.

For men, the prevalence of polypharmacy (DP≥5) increased in all age groups (11.9%) except for the age group 0-9 years. The largest increase in the prevalence of polypharmacy was in the age group 60-69 with an increase of 12.3%, whilst in the age groups 70-79 to 90-years, the increase was between 8.4% and 10.1% (Figure 2).

For women, the prevalence of polypharmacy (DP≥5) increased in all age groups (5.9%) except for the age group 0-9 years. The largest increase in the prevalence was in the age group 10-19 with an increase of 13.3%, whilst in the age groups 60-69 to 90-years, the increase was between 5.8% and 7.6% (Figure 2).

### The development of excessive polypharmacy

The prevalence of excessive polypharmacy (DP≥10) in the entire population increased by 15.7% (from 0.021 to 0.024) 2005-2008 (Table 1). The number of individuals with DP≥10 increased by 18.1% (from 185,618 to 219,244) (Table 2).

The level of prevalence increased in all age groups except for the age group 0-9 years and the largest increase in the prevalence was in the age group 90-with an increase of 28.5%. In the age groups 60-69 to 80-89 years, the increase was between 10.6% and 21.6% (Figure 1).

**Figure 3:**
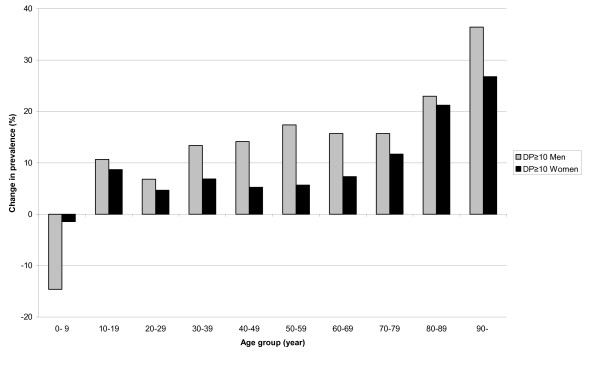
**The change in prevalence of excessive polypharmacy for men and women**. The change (%) in prevalence of excessive polypharmacy (DP≥10) for men and women in different age groups in Sweden 2005-2008.

For men, the prevalence of excessive polypharmacy (DP≥10) increased in all age groups (20.2%), except for the age group 0-9 years. The largest increase in the prevalence was in the age group 90-, with an increase of 36.4%. In the age groups 60-69 to 80-89 years, the increase was between 15.7% and 23.0% (Figure 3).

For women, the prevalence of excessive polypharmacy (DP≥10) increased all age groups (13.5%), except for the age group 0-9 years. The largest increase in the prevalence was in the age group 90-with an increase of 26.8%, and in the age groups 60-69 to 90-years, the increase was between 7.3% and 21.2% (Figure 3).

### The development of the mean number of dispensed drugs per individual

The mean number of dispensed drugs per individual, during a 3-month period for all individuals in Sweden receiving dispensed drugs, increased by 3.6% (from 3.3 to 3.4 drug per individual) during the study period 2005-2008.

For elderly individuals, 70 years and above, the mean number of dispensed drugs per individual increased by 3.9% (from 4.8 to 5.0 drugs), by 6.1% (from 5.7 to 6.1 drugs), and by 7.6% (from 6.1 to 6.6 drugs), in the respective age groups. The increase (%) of the mean number of dispensed drugs for men and for women was similar.

## Discussion

### Principal findings and possible explanations

The prevalence of polypharmacy and excessive polypharmacy increased year-by-year, in the entire Swedish population 2005-2008.

With the exception of the age group 0-9 years, the prevalence of polypharmacy and excessive polypharmacy increased in all age groups. The prevalence of excessive polypharmacy displayed a clear age trend, with the largest increase for the age groups 70 years and above. Generally, the increase in the prevalence of polypharmacy was approximately twice as high for men as for women, and the increase in prevalence of excessive polypharmacy was about 1.5 as high for men as for women.

The increase rate for both polypharmacy and excessive polypharmacy levelled out during the study period, but between separate years, we noted a variation in rate of increase. This variation refers to the different age groups and to both genders.

The increase in the prevalence of polypharmacy may have several different causes: changes in the recommended prescriptions for various drug treatments as well as the introduction of specific drugs for treatment of conditions/diseases regarding which they have previously not been applied. Furthermore, middle-aged individuals are increasingly informed, and become, consequently, more prone to request an increased amount of prescription drugs. Finally, more drugs are being prescribed for preventive use. All together, these factors may have resulted in a change in the physicians' prescription patterns.

The decrease in the prevalence of polypharmacy in the age group 0-9 years can be explained by the national interventions to reduce the prescribing of antibiotics to children, in order to prevent antimicrobial resistance. Nearly 80% of the children 0-9 years with polypharmacy received antibiotics in 2006, clearly indicating that antibiotics have the largest impact on the prevalence of polypharmacy in this particular age group [15].

Both the overall increase and the differences in the rate of increase between the years are puzzling. These increases suggest relatively rapid changes in prescription patterns among prescribers; changes that may have a variety of causes, e.g. the introduction of new clinical guidelines.

Prior to 2005, national clinical guidelines were available for only three different areas in Sweden. During the study period, 2005-2008, The National Board of Health and Welfare in Sweden introduced four new national clinical guidelines; Stroke, Chest-Colorectal cancer and Prostate cancer, Heart disease, and Addiction, and in 2009-2011 seven other new clinical guidelines are planned to be introduced (e.g. Depression, Dementias, Diabetics, Lung cancer).

Prior to being officially introduced, new clinical guidelines exist only in preliminary versions. Consequently, these guidelines might influence the prescription habits and the development of polypharmacy a number of years before the guidelines being officially introduced. The introduction of national clinical guidelines for heart diseases and prostate cancer might explain both the unequal increase between genders, and the variation in increase rate between the different years.

In a study from Sweden concerning general practitioners' (GPs') perceptions of multiple-medicine use [18], clinical guidelines were viewed as "medicine generators". GPs' expressed frustration concerning guideline recommendations for certain diagnoses, e.g. cardiovascular diagnoses that "immediately result in five medicines". Regardless of the patients' other diseases, many guidelines were perceived as too rigid, leading to a standard "kit" of medicines per indication, and thereby resulting in that individuals with multiple diseases received an increasing number of different drugs.

The introduction of new national guidelines might therefore also contribute to explaining the age trend in the development of excessive polypharmacy, as older patients are more often exposed to several diseases. The elderly may receive, as a result of the guidelines, more often than others, a number of different "kits" of drugs added [18].

### Strengths and weaknesses of the study

Our study presents an overview of the development of polypharmacy in an entire national population. The applied 3-month period prevalence of dispensed drugs includes all drugs that are prescribed on a regular basis (e.g. drug used in diabetes), when needed (e.g. analgesics), and temporarily (e.g. antibiotics). The periodically used drugs have been shown to have a different impact on the prevalence of polypharmacy in different age groups [15].

As the study included all individuals in the population, we avoided certain known problems concerning sampling, recall, interview and confidence. On the other hand, when the register data regarding the dispensed drugs is used as an estimator of drug use and polypharmacy, over-as well as underestimations of actual drug use arises. The extracted data included dispensed prescription drugs only, corresponding to approximately 82% of all Defined Daily Doses (DDD) distributed in Sweden. Also, additional sources of drugs, such as OTC medications, in-hospital medications and non-institutional care medications, herbal and alternative remedies together with previously filled prescriptions (before the study period), gifts and elicit Internet sales, were not included in the study, and resulted in an underestimation of the total consumption of drugs.

In addition, generic duplication (intended and unintended duplication of dispensed drugs with the same substance) might also have caused an underestimation of polypharmacy in our data, as we calculated only the number of dispensed drugs comprised of different substances. In sample studies of drug use among individuals with polypharmacy, patients often have two or more drugs with the same substance [4,19,20]. In register studies, it is difficult to make distinction between generic duplication and generic substitution (an intended switch between two drugs with the same substance). If the generic duplicate had been taken into account, this would have resulted in an even larger prevalence of polypharmacy. Whether the generic duplicate could have any impact on the development of the prevalence of polypharmacy over the study period has not been addressed.

Conversely, dispensed drugs as an indicator of drug use might result in an overestimation, as it is well known that a certain proportion of all dispensed drugs will never be used [21].

### Strengths and weaknesses in relation to other studies

The displayed increase of polypharmacy in the entire population in Sweden since 2005 is in line with studies focusing only on elderly individuals during the 1980's and 1990's [2,22-25].

However, there are certain difficulties in comparing our results concerning the elderly population with some of the previous studies. Firstly, some studies have addressed the level of drug use for the same individuals over time, concluding that drug use and polypharmacy increase with increasing age, but without an increased prevalence over time [26-30].

Secondly, some studies have applied varying time periods, different definitions of drug use and polypharmacy or different samplings of individuals [3]. Finally, certain studies are based on interviews, and their results might be influenced by the sampling, recall or interview bias impedes comparison with results from register-based studies [15,31].

The displayed year-by-year increase in drug use, polypharmacy and the mean number of dispensed drugs in the present study is generally minor compared to the increase shown in previous studies of the development of drug use in the 1980 s and 1990 s, e.g. a displayed 3-fold increase in the prevalence of polypharmacy and mean number of drugs per person during a ten year period [2]. This difference might be explained by the fact that our data included all individuals in the national population. Previous studies have often used samples of only the elderly admitted to hospitals or living in nursing homes. Relatively healthy individuals might, therefore, not have been included in these earlier studies. Another possible explanation is that the recent efforts to reduce the increases in drug use and polypharmacy actually have had an effect.

### Implications for clinicians and policymakers

The substantial increase in the prevalence of polypharmacy and excessive polypharmacy occurs simultaneously with the introduction of new clinical guidelines aimed at increasing the benefits of the medical treatment. The increase also occurs when the potential risks with polypharmacy have been highlighted, and various efforts have been made to reduce the number of drugs prescribed to individuals with an excessive number of drugs, especially the elderly. In Sweden, efforts to reduce the prevalence of polypharmacy have been focused on, at in first hand, the reduction of unintended generic duplication.

The assessment of the increasing prevalence of polypharmacy is not interpreted in a unanimous manner. For certain clinicians and policymakers, the results of the present study may be interpreted as the regrettable further development of polypharmacy, and that, in particular, excessive polypharmacy is continuing in an undesirable direction. However, the results of our study may also be interpreted to imply that a larger proportion of patients are receiving recommended drug treatment in line with new clinical guidelines.

The prevalence of polypharmacy may hide the fact that the benefits and/or risks of polypharmacy can be evaluated at individual level only. For clinicians, recommendations are required as to the manner in which to combine and balance different clinical guidelines to achieve an appropriate drug therapy for patients with multiple diseases.

### Unanswered questions and future research

Over the 4-year study period, the increase in the prevalence of polypharmacy and excessive polypharmacy was particularly notable for men, 12% and 20%, respectively, and was even more notable for elderly men. This increase in drug use remains to be analyzed, and can possibly be associated with the introduction of new national clinical guidelines during the period with special relevance for men (e.g. guidelines for Heart diseases and Prostate cancer).

## Conclusions

The prevalence of polypharmacy and excessive polypharmacy, as well as the mean number of dispensed drugs per individual, increased year-by-year in Sweden 2005-2008.

## Competing interests

The authors declare that they have no competing interests.

## Authors' contributions

All authors participated in the design of the study and the discussion of findings. BH and KH executed the data management and BH drafted the manuscript. KH, BÅ and GP revised the manuscript. All authors read and approved the final manuscript.

## Pre-publication history

The pre-publication history for this paper can be accessed here:

http://www.biomedcentral.com/1472-6904/10/16/prepub
